# Harnessing the ECM Microenvironment to Ameliorate Mesenchymal Stromal Cell-Based Therapy in Chronic Lung Diseases

**DOI:** 10.3389/fphar.2021.645558

**Published:** 2021-04-15

**Authors:** Linda Elowsson Rendin, Anna Löfdahl, Måns Kadefors, Zackarias Söderlund, Emil Tykesson, Sara Rolandsson Enes, Jenny Wigén, Gunilla Westergren-Thorsson

**Affiliations:** Lung Biology Unit, Department of Experimental Medical Science, Lund University, Lund, Sweden

**Keywords:** chronic objective pulmonary disease, MSC, extracellular matrix, artificial lung scaffolds, idiopathic pulmonary fibrosis

## Abstract

It is known that the cell environment such as biomechanical properties and extracellular matrix (ECM) composition dictate cell behaviour including migration, proliferation, and differentiation. Important constituents of the microenvironment, including ECM molecules such as proteoglycans and glycosaminoglycans (GAGs), determine events in both embryogenesis and repair of the adult lung. Mesenchymal stromal/stem cells (MSC) have been shown to have immunomodulatory properties and may be potent actors regulating tissue remodelling and regenerative cell responses upon lung injury. Using MSC in cell-based therapy holds promise for treatment of chronic lung diseases such as idiopathic pulmonary fibrosis (IPF) and chronic obstructive pulmonary disease (COPD). However, so far clinical trials with MSCs in COPD have not had a significant impact on disease amelioration nor on IPF, where low cell survival rate and pulmonary retention time are major hurdles to overcome. Research shows that the microenvironment has a profound impact on transplanted MSCs. In our studies on acellular lung tissue slices (lung scaffolds) from IPF patients versus healthy individuals, we see a profound effect on cellular activity, where healthy cells cultured in diseased lung scaffolds adapt and produce proteins further promoting a diseased environment, whereas cells on healthy scaffolds sustain a healthy proteomic profile. Therefore, modulating the environmental context for cell-based therapy may be a potent way to improve treatment using MSCs. In this review, we will describe the importance of the microenvironment for cell-based therapy in chronic lung diseases, how MSC-ECM interactions can affect therapeutic output and describe current progress in the field of cell-based therapy.

## Introduction

Chronic lung diseases such as idiopathic pulmonary fibrosis (IPF) and chronic obstructive pulmonary disease (COPD) are life-threatening progressive lower respiratory diseases that are increasing world-wide. COPD alone is the 3^rd^ cause of death, and complicated by co-morbidities such as cardiovascular disease and lung cancer (Sin et al., 2006). COPD patients are mainly treated with bronchodilators and inhaled corticosteroids to help reduce symptoms, however, disease progression is not halted (Yawn et al., 2021). The complex pathology exhibits a diverse spectrum of phenotypes with formation of fibrosis and/or emphysema and current treatment options involve only strategies to slow down the disease progression, and to improve quality of life (Negewo et al., 2015; Barrecheguren and Miravitlles, 2016). IPF, affecting 3 million people world-wide (Martinez et al., 2017) is also associated with cardiovascular comorbidities (Caminati et al., 2021) and share the risk factors of smoking and inhaled toxins with COPD. IPF, being a chronic fibrosing interstitial lung disease (ILD) with unknown etiology, is characterized by the histopathological pattern of usual interstitial pneumonia (UIP). The abnormal wound healing in response to epithelial distress results in exaggerated mesenchymal cell activities including build-up and turnover of ECM, causing the altered biomechanics and increased stiffness of the lung tissue. This ultimately disrupts gas exchange and lung function (Raghu et al., 2011). No treatment today affects the mortality associated with IPF to a great extent, but merely prolongs life, with patients still rarely exceeding 3–5 years after IPF diagnosis (Raghu et al., 2011). IPF is primarily treated with the antifibrotic drugs Nintedanib or Pirfenidone, which generate prolonged life-span, but treatment adherence is unfortunately low due to multiple side effects. Future treatment strategies include combining drugs to manage several activated cellular pathways that may differ between patients (Gao et al., 2021). Therefore stratifying patients in more detail may uncover novel therapeutic targets, which is reviewed in Trachalaki et al. (Trachalaki et al., 2021). The lack of curative treatments for both IPF and COPD therefore opts for new efficient strategies that not only affect disease severity but also inhibit progression or at best reverse the deterioration entirely and healing the lung. This is a priority for individual patients as well as for society (Hilberg et al., 2018), and the socioeconomic costs due to these diseases could be significantly reduced by minimizing prevalence and severity (Anees Ur and et al., 2020).

**FIGURE 1:**
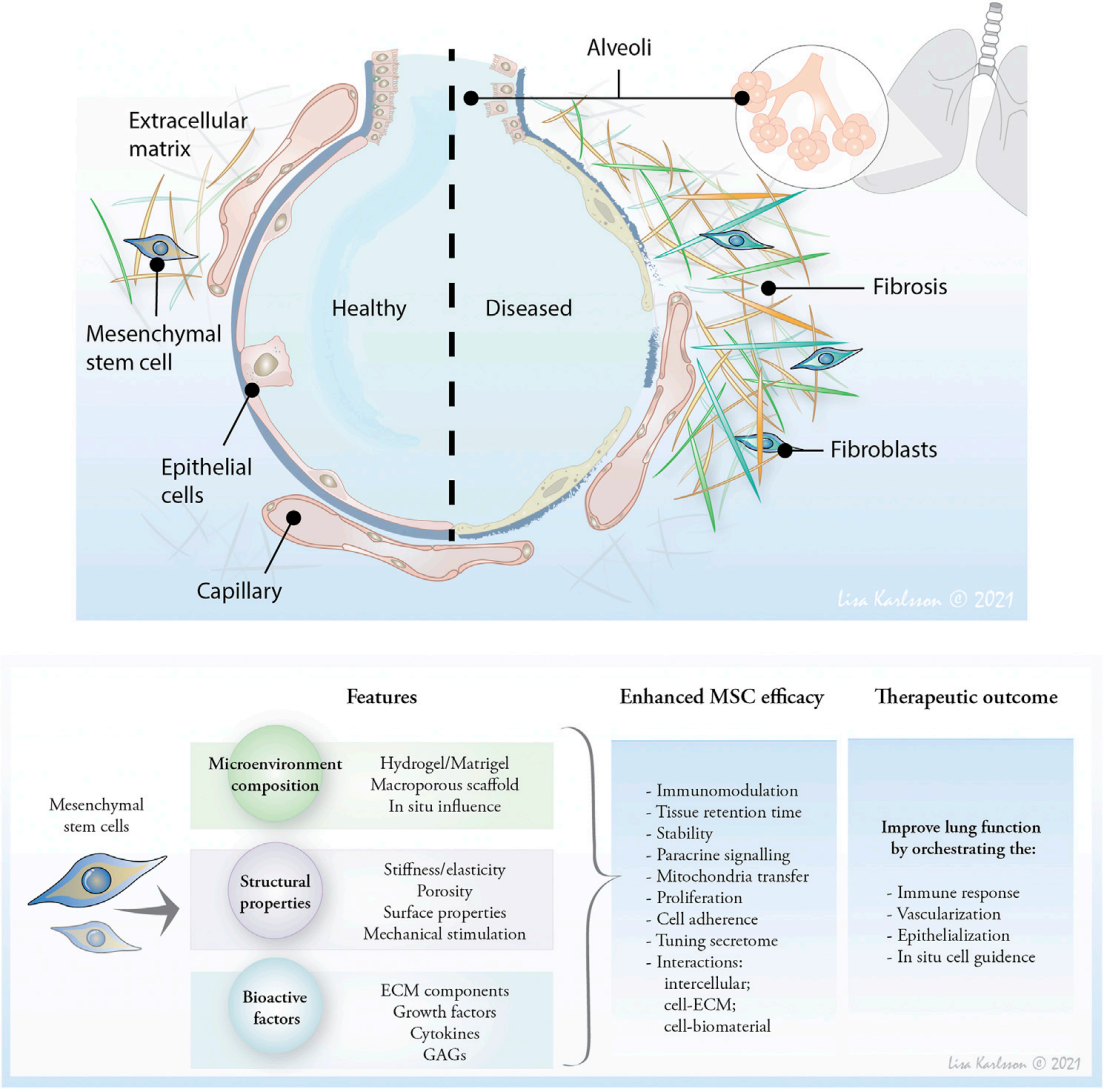
The composition and mechanoelastic properties of ECM affect cell response and activity and therefore, moderating the microenvironment of therapeutic mesenchymal stem cells (MSC) may provide a novel approach to affect cell retention time, and improve therapeutic properties. Affecting the ECM *in situ* or providing an artificial microenvironment to enhance MSC therapeutic potential are future lines of research to affect the distorted lung tissue and reduce lung inflammation, and optimally heal emphysematic and fibrotic lesions to restore oxygen uptake.

Common to both diseases is the structural remodeling of the lung tissue, resulting in impaired gas exchange that leads to decline in lung function. To date, the knowledge of the inherent capacity of the lung to repair is limited, but mesenchymal stromal cells (MSC) have been proposed to have a therapeutic potential due to their regenerative and immunomodulatory abilities in combination with their low immunogenicity attributed to low or absent human leukocyte antigen (HLA) expression (Lee et al., 2014). The structurally remodeled ECM may have a larger impact on cellular behavior in the local microenvironment than previously anticipated. An altered ECM actively affects the biomechanical properties of the lung tissue (Elowsson Rendin et al., 2019) and its function, creating an inherent vicious feedback loop that further propagate these diseases (Figure 1). Therefore, approaching the microenvironment of the lung to control local pathological cell responses that may also enhance therapeutic activities of local MSC, is an appealing therapeutic thought that can revolutionize treatment of serious chronic lung diseases such as IPF and COPD. This review elaborates on ways to modulate the pulmonary microenvironment of the therapeutic MSCs to improve and control efficacy of MSC treatment. This could mean providing an injectable cell-instructive artificial milieu (scaffold), or by directly affecting *in situ* environment of the injected MSC.

## MSC Based Therapy in IPF and COPD

MSCbased therapy has been explored in clinical trials in both IPF and COPD using intravenous (i.v.) or intratracheal routes of administration of bone marrow, umbilical cord or adipose tissue derived MSC in a dosage range of 10^6^–10^8^ cells per dose, single dose or in some cases, up to 4 doses. Completed trials using primarily one dosage regimen show in COPD trials a safe profile and ameliorated clinical parameters in up to 50% of patients (Cruz and Rocco, 2020). In a small human trial in IPF, patients demonstrated that allogeneic MSC treatment is safe and tolerable upon applying a single injected dose of bone marrow derived MSC up to 200 x 10^6^ cells (Glassberg et al., 2017). Similarly, low adverse effects were also shown when using placenta derived MSC (i.v. infusion) or adipose derived stromal cells from stromal vascular fraction (endobronchial infusion) in a phase 1b study in IPF patients (Tzouvelekis et al., 2013; Chambers et al., 2014). A recent study examined the efficacy of repeated i. v. administered MSC, showing improved lung function and 6°min walk test in patients with moderate to severe IPF(Averyanov et al., 2020). Collectively, pre-clinical studies demonstrate antifibrotic effects with MSC treatment, however, the long-term beneficial effects and feasibility of MSC as a therapeutic strategy in IPF patients are yet to be determined, where the few existing clinical trials demonstrate an acceptable safety profile. According to ClinicalTrials.gov, there are currently 5 clinical trials recruiting for stem cell treatment of pulmonary fibrosis. Of these, one is carried out at the University of North Carolina, Chapel Hill, United States, with the aim to evaluate safety and efficacy of infusing autologous lung spheroid stem cells derived from trans-bronchial biopsy specimens in IPF patients.

Several animal studies show promising results in treating pulmonary fibrosis with MSC or derivates thereof such as exosomes and extracellular vesicles (EVs), alleviating fibroblast activation and ECM deposition along with anti-inflammatory outcomes (Reddy et al., 2016; Mansouri et al., 2019; Gad et al., 2020; Wan et al., 2020). Pre-clinical studies on COPD demonstrate that MSCs reduce inflammation and improve histological lung structure (Cruz and Rocco, 2020), which have been translated into several human clinical trials for treatment of COPD(Ribeiro-Paes et al., 2011; Weiss et al., 2013; Stolk et al., 2016; de Oliveira et al., 2017; Armitage et al., 2018). These clinical trials all had safety as their primary endpoint and was not powered for detection of efficacy. Although the clinical trials failed to reproduce the promising pre-clinical results, they all demonstrated that MSC administration was well tolerated among COPD and emphysema patients (Ribeiro-Paes et al., 2011; Weiss et al., 2013; Stolk et al., 2016; de Oliveira et al., 2017; Armitage et al., 2018). Interestingly, Armitage et al. demonstrated that MSCs radiolabeled with indium-111 reached the lungs within 30 min and remained detectable for 24 h after administration (Armitage et al., 2018). This should mean that the effect of MSCs on damaged lung tissue and resident cells need to be rapid. Moreover, an increasing body of research suggests that the MSC therapeutic functions depend on the microenvironment encountered (Kusuma et al., 2017; Abreu et al., 2019; Islam et al., 2019; Abreu et al., 2020). Therefore, it is tempting to speculate that by modifying the microenvironment it would be possible to enhance the therapeutic time-frame for MSCs by increasing residency in the COPD lungs and thereby increasing the potency of the infused MSCs. Currently, there are three clinical trials recruiting patients to evaluate MSC for the treatment of COPD. A comprehensive overview of ongoing and completed human trials in IPF and COPD is searchable at ClinicalTrials.gov. These trials are encouraging and gives great hope to the scientific community to increase the therapeutic potential of MSC.

## The Mechanism of MSC

### Anti-Inflammatory Function

MSCs are extensively explored as cell therapy candidates and are known for their potential to differentiate into multiple cell lineages, however their major therapeutic mechanism is from interactions via paracrine signaling and cell-cell contact (Murphy et al., 2013; Gao et al., 2016). MSCs have demonstrated both anti-inflammatory and anti-fibrotic capacity, which could be of importance for tissue repair and regeneration in chronic lung diseases (Cruz and Rocco, 2020). The observed anti-inflammatory properties of MSCs include effects on several cells of the immune system, including T-cells, B-cells, dendritic cells, monocytes, macrophages, and natural killer (NK) cells. For example, MSCs can suppress T-cell proliferation via Indoleamine-2,3dioxygenase (IDO) secretion, which mediates tryptophan depletion causing cellular stress in T cells (Meisel et al., 2004; Laing et al., 2019), or by inducing a switch toward a regulatory T-cell phenotype (Bartholomew et al., 2002; Benvenuto et al., 2007; Casiraghi et al., 2008). In an inflammatory setting, MSCs are able to direct macrophage polarization from a proinflammatory (M1) to an anti-inflammatory phenotype (M2). This occurs through feedback-loop involving MSC secreted prostaglandin E2 (PGE2) (reviewed in) (Prockop, 2013). In addition, MSCs are able to affect resident macrophages to reduce their secretion of pro-inflammatory mediators through a different negative feedback-loop, by secreting tumor necrosis factor (TNF)-α stimulated gene 6 (TSG-6) (Prockop, 2013).

While the anti-inflammatory effect of MSCs has been extensively used as a rationale for MSC-based therapy, their anti-fibrotic qualities could further motivate the use of MSC-based therapy in chronic diseases. In animal models of lung fibrosis, it has been shown that MSC administration can reduce the extent of fibrosis (reviewed in (Srour and Thébaud, 2015). Increased levels of the hepatocyte growth factor (HGF) has been linked to the anti-fibrotic properties of MSCs. *In vitro*, HGF inhibited epithelial-mesenchymal-transition (EMT) through inhibition of transforming growth factor β (TGF-β) signaling (Mias et al., 2009; Shukla et al., 2009). Interestingly, administration of MSC has been associated with changes in levels of ECM modifying enzymes such as metalloproteases (MMPs) and tissue inhibitor of metalloproteases (TIMPs), and the balance of these enzymes is important in both fibrosis and emphysema development (Mias et al., 2009; Moodley et al., 2009; Semedo et al., 2009).

### Operating Neighboring Cells: Extracellular Vesicles

In addition to the secretion of anti-inflammatory and anti-fibrotic mediators, MSCs are also known to communicate with other cells by secretion of EVs (reviewed in (Farhat et al., 2019)). EVs are a heterogenous group of small lipid vesicles with cargoes containing mediators for intercellular communication such as proteins, microRNAs, and lipids (Thery and et al., 2018; van Niel et al., 2018). An increasing body of literature suggests that MSC-derived EVs possess immune regulatory functions (Farhat et al., 2019). MSC-derived EVs have been demonstrated to mitigate allergic hyperresponsiveness (Cruz et al., 2015), enhance M1 to M2 macrophage phenotype polarization, macrophage bioenergetics, and phagocytosis (Phinney et al., 2015; Jackson et al., 2016; Morrison et al., 2017), as well as to have an inhibitory effect on T-cell proliferation (Blazquez et al., 2014; Pachler et al., 2017; Mardpour et al., 2019). Moreover, MSC-derived EVs have been shown to decrease the influx of inflammatory cells in an *E. coli* endotoxin-induced acute lung injury model. This therapeutic effect was at least partly mediated through keratinocyte growth factor (KGF/FGF7)(Zhu et al., 2014). Interestingly, in an *E. coli*-induced acute lung injury rat model, it was demonstrated that EVs isolated from MSCs pre-exposed to IFN-g were more effective in attenuating lung injury compared to EVs isolated from naïve, non-exposed MSCs (Varkouhi et al., 2019). These data suggest that the EV cargo released by MSCs can vary significantly based on the microenvironment the cells have encountered.

### Restore Cellular Function: Mitochondrial Transfer

Another possibility of MSCs potential in cell therapy is to use their mitochondria. The first evidence that MSCs have the capacity to transfer mitochondria to other cells was published in 2006 (Spees et al., 2006). In this study MSCs were able to restore the aerobic respiration in epithelial cells lacking functional mitochondria (Spees et al., 2006). Since then, it has been shown that MSCs can transfer mitochondria via EVs, microtubules, and tunneling nanotubes (TNTs) (Spees et al., 2006; Phinney et al., 2015; Morrison et al., 2017). For example, mitochondria transferred from MSCs to macrophages partly via TNTs, modulated macrophage phagocytosis both *in vitro* and *in vivo* (Jackson et al., 2016). MSC mitochondrial transfer has also been reported to reduce lung injury in a cigarette smoke-induced emphysema model (Li et al., 2014). Furthermore, Islam et al. reported that MSCs could protect against acute lung injury through mitochondrial transfer via Cx43-dependent alveolar attachment (Islam et al., 2012). Interestingly, Mahrouf-Yorgov et al. observed that MSCs had the capacity to sense, and subsequently engulf and degrade mitochondria from damaged cells. This process enhanced mitochondrial biogenesis and mitochondrial transfer by the MSCs (Mahrouf-Yorgov et al., 2017). Taken together, future directives using MSC based therapy in chronic lung diseases needs to consider the engraftment in the lung for therapeutic efficacy (Behnke et al., 2020).

## The Intrinsic Properties of the Lung-ECM

MSCs and other mesenchymal cells are the main producer of ECM. Interestingly, the role of the ECM is receiving increasing recognition as an important element in controlling cellular behavior during homeostasis and in disease progression (Deng et al., 2020). Apart from providing mechanical and structural support, the complex 3D structure of the ECM, consisting of glycoproteins, collagens, glycosaminoglycans (GAGs), proteoglycans, dictate cell migration, polarity, proliferation, differentiation, and survival, where the dynamics of the ECM molecules play a critical part (Wigén et al., 2019). Some proteins e.g., elastin fibers are stable over almost a lifetime (Shapiro et al., 1991), while certain collagens have a short turnaround, changing every day (Laurent, 1987). In the pulmonary interstitial layer, the major structural components are fibrillar collagens type I, III, IV, V, and XI (extensively reviewed by Laurent, 1987; Sherman 2015) that make up the core structure and contribute to the tensile strength, and elastin along with fibrillin contribute to the compliance of the tissue (Laurent, 1987; Sherman et al., 2015). This intricate meshwork of fibers is also composed of glycoproteins, GAGs, and proteoglycans providing the tissue with its viscoelastic properties. In the alveolar space, the epithelial and endothelial cells are anchored to a basement membrane primarily consisting of collagen type IV, laminins, and proteoglycans. Both in IPF and COPD the basement membrane and the interstitial layer are affected with altered structural properties affecting the biomechanics. In IPF, the alveolar basement membrane is fragmented and the interstitial layer is thickened, while in COPD, the ECM components are degraded and the structural properties are lost (Burgess et al., 2016)

### Glycosaminoglycans and Growth Factors

While the most studied group of ECM molecules are the various collagens, lately however the family of proteoglycans have drawn large attention. Proteoglycans are proteins decorated with covalently attached linear polysaccharides, GAGs. The proteoglycans are heterogeneous molecules-both in terms of the core protein structure, as well as the chain length and chemical structure of the GAG side chains. These side chains strongly decide the physical properties of the proteoglycans. The main classes of proteoglycan-bound GAGs in the human lung are heparan sulfate/heparin (HS/hep) chondroitin/dermatan sulfate (CS/DS) and keratan sulfate (KS) (Papakonstantinou and Karakiulakis, 2009). A fourth type of GAG, hyaluronan (HA), is also prevalent in the lung, but is not bound to a core protein (Couchman and Pataki, 2012). All four types of GAGs contain disaccharide motifs, which are repeated throughout the polymer. For HS/hep, CS/DS and KS the disaccharide units can be modified by sulfation at various positions, giving rise to motifs/domains (Persson et al., 2020), which in turn are important for binding of specific mediators to be involved in chronic lung diseases such as TGF-b, HGF, and TNF-a (Esko et al., 2015). In this way the GAGs play an important role in interacting with growth factors, where the GAGs build gradients during embryonic development but also during tissue repair and delivering of growth factors to receptors. Interestingly, alterations in sulfation pattern, or induction of deacetylations and epimerizations in the GAG chains affect their role in tuning growth factor gradients, which alter cell activities (Wigén et al., 2019). In IPF, an increase in sulfation of both HS and CS/DS has been recognized in the border zone toward fibrotic lesions (Westergren-Thorsson et al., 2017). This may reflect an altered pulmonary landscape, navigating nearby cells toward regions of activated repair process, promoting a profibrotic phenotype.

### ECM Bound Growth Factors

Arguably one of the most important interactions between cells is mediated through growth factors and cytokines. These factors travel large distances in the body to recruit and direct cells where they are needed. A largely overlooked process is how growth factors interact with the ECM via GAGs and how the ECM can increase or decrease the cell response to growth factors. Thus, the ECM serves as a reservoir for growth factors, which are slowly released over time. At the same time the growth factors bound to the ECM via the GAGs become protected from circulating proteases. Because of these properties, if the ECM is injured the release of growth factors will be instant, thus triggering an immediate cell response. For example, latent TGF-β (Saito et al., 2018) in the ECM become activated upon injury and is known to be one of the factors activating mesenchymal cells that in fibrosing diseases such as IPF lead to an over production of ECM. Another example of ECM bound factor is fibroblast growth factor (FGF)-2, which binds on a nanomolar level to HS (Ibrahimi et al., 2004). This GAG-growth factor complex increases the binding to the FGF receptor 1 and is crucial for adequate cell signaling (Yayon et al., 1991) and may be immunomodulatory factor in COPD (Tan et al., 2020). Therefore, the ECM and growth factor complexes works to fine-tune cell responses and control timing, making it an important target in the development of therapies in chronic lung diseases.

### Cell-ECM Interaction

Even though lung cells derived from patients with chronic lung disease have shown to have inherent cellular features such as gene expression and proteomic profile (Hallgren et al., 2010; Woldhuis et al., 2020), the microenvironment of the tissue, however, appears to be the dominating factor in regulating cellular behavior, at least *in vitro* (Elowsson Rendin et al., 2019). It has been demonstrated that the pathological reconstructed ECM in IPF overrules intrinsic cellular characterizations, ultimately repr ogramming cells for fibrogenesis (Parker et al., 2014; Philp et al., 2018; Rodriguez et al., 2018). The wavy and aligned collagen structures of the ECM in distal IPF tissue alter migration patterns and cell morphology, creating elongated human lung fibroblasts with increased migratory speed (Tisler et al., 2020). Lung fibroblasts engrafted on fibrotic or emphysematous lung tissue readily adapt to surrounding structures and ECM niches of the lung with high morphological plasticity (Burgstaller et al., 2018). Non-cultured fibroblasts, isolated from lungs of IPF patients, demonstrated an altered genomic profile in comparison to healthy fibroblasts (Emblom-Callahan et al., 2010), as do epithelial cells when cultured on bronchial-ECM derived from COPD (Hedström et al., 2018). The positive feedback-loop created between cells and ECM is manifested quickly with marked differences in cellular activity, inducing a shift in the production of ECM proteins that steers the cellular response toward a continuous rebuild of fibrotic tissue with altered basement membrane structures (Elowsson Rendin et al., 2019). The constant changes in ECM composition during disease progression, resulting in pathological differences in lung architecture creates a disease specific proteomic change where the local microenvironment signals for continuous tissue reconstruction (Westergren-Thorsson et al., 2017; Åhrman et al., 2018).

The deformed lung tissue, caused by either excessive tissue repair or the lack thereof, also has an immense impact on the mechanical properties of the tissue, where mechanical forces sensed by surrounding cells activate mechanosensitive pathways (e.g., YAP/TAZ) and influence cell behavior (Asano et al., 2017; Haak et al., 2018). To maintain tissue homeostasis, the cells require anchoring to the ECM via integrin binding through which the cells sense the microenvironment and activate signaling transduction pathways, inducing mechanical and chemical intracellular signaling through the actin cytoskeleton (reviewed by Vogel, 2018 (Vogel, 2018)). Changes in the local mechanical properties create a mechanical gradient that affects cellular behavior such as migration, proliferation and differentiation. Integrins are also thought to be involved in growth factor signaling, acting synergistically with receptors for different growth factors. More specifically, the integrins are known to bind to Arg-Gly-Asp (RGD) motifs found predominantly on fibronectin (FN), but also in ECM proteins e.g., laminin, tenascin, and vitronectin (Widhe et al., 2016; Miron-Mendoza et al., 2017). Integrins are also the main receptors forming focal adhesions and are thought to be required for cell motility. The local microenvironment has been found to be decisive for the size and concentration of focal adhesions, which further impact on cell behavior (Wozniak et al., 2004; Costa et al., 2013).

## Modulating the 3D Environment for Treatment

There are several *in vitro* studies showing how MSC are differently affected by the environment the cells encounter. Increasing research is focused on how to control the therapeutic effect of MSC, where one option being explored is by modulating the microenviroment *in situ* or by injectable biomaterials.

### Hydrogel Encapsulation

Encapsulating MSC in injectable hydrogels is extensively explored to increase retention time and to modulate the cellular activity *in situ* (Farhat et al., 2019; Wong et al., 2020) by providing the cells with a 3D microenvironment that protects and promotes an anti-inflammatory and regenerative response, see outline in Figure 1. Techniques range from single cell encapsulation to larger scaffolds with macroporous structure (Kim et al., 2019). Efforts have been made to mimic the mechanical properties of the ECM to steer cellular behavior in health and disease (de Hilster et al., 2020; Pouliot et al., 2020). So far, hydrogels have been by far the most produced and studied substrate, where ECM-based hydrogels from murine, porcine and human tissues have been developed with different decellularization methods. In order to mimic the natural tissue when designing an artificial matrix, it is vital to maintain the components of the ECM, often measured as the GAG-DNA content ratio, as well as the viscoelastic properties (Uhl et al., 2020). Apart from hydrogels based on decellularized tissue, there are various natural, synthetic and hybrid (meaning combining natural, synthetic or ECM components) polymers that are being explored, including proteins, polysaccharides, recombinantly expressed peptides, and polyethylene glycol (Uhl et al., 2020). The advantage of hydrogels is that the structural properties can be easily modified through the crosslinking of the hydrophilic polymer chains. In addition, the biomaterial can be modulated by incorporating cell-instructive factors e.g. growth factors, RGD domains, and ECM proteins. Recombinantly produced biomaterials such as the elastin-like recombinamer polymer allows for the precise composition of structural and chemical properties, with gene technology introducing sequences for cell attachment and MMP degradation and at the same time exposing functional groups for binding of for example growth factors to promote or maintain the MSC therapeutic effect (Ibáñez-Fonseca et al., 2020). De Santis et al. recently demonstrated the functional outcome of a 3D bioprinted hybrid hydrogel combining decellularized lung tissue with alginate to form human airways containing primary human airway epithelial progenitor cells (De Santis et al., 2020). Transplanted in mice, the cells showed evidence of differentiation into mature epithelial cells. The hydrogel properties both had matched biomechanical properties and contained ECM instructive factors from the native lung tissue (De Santis et al., 2020). Hydrogels based on collagen type I, one of the major structural components of tissues, has been extensively explored in combination with other materials such as silk, a highly elastic protein, to improve the mechanical properties to mimic the viscoelastic properties of tissues (Sanz-Fraile et al., 2020). As discussed above, GAGs multiple functions in cell signaling and in tissue homeostasis and have been explored as biomaterials. CS that have modifiable functional groups for covalent and non-covalent bonding has been used for the development of injectable hydrogels to tune for different physical properties as well as release of therapeutic factors (Ornell et al., 2019).

### Macroporous Scaffolds

In contrast to solid hydrogels, macroporous biomaterials or scaffolds introduces a more complex 3D environment, where the surface properties of the scaffold can be tuned to functionalize cell attachment, differentiation and migration, as well as to enhance cell diffusion to the scaffold.

In a recently published article, a collagen-based scaffold was developed with macroporous structures to mimic the distal lung tissue. The scaffold was functionalized with collagen binding HGF to enhance the regeneration of alveolar-like structures (Wang et al., 2020). When making a porous scaffold, pore size is often determined by changing the initial concentration of the reagents, which in turn changes the bulk stiffness. By adding an antifreezing agent (e.g., DMSO), pore size can be kept constant regardless of the initial concentration (Jiang et al., 2019). Jiang et al. could show that larger and stiffer pores induced an anti-inflammatory phenotype of macrophages (Jiang et al., 2019). Shamskhou et al. has shown that local delivery of IL-10 using a hyaluronan and heparin-based hydrogel system had an anti-inflammatory effect in bleomycin-treated mice (Shamskhou et al., 2019).

### Pre-Conditioning of MSC to Enhance Therapeutic Effect

There are increasing evidence that MSC have a cellular memory, with sustained responses to preceding stimuli, which may influence the desired effect of MSC-based therapies (Yang et al., 2014). MSC cultured on substrates with different ECM molecules have been shown to affect the paracrine function of MSC. For example, De Lisio et al. showed that cell cultures on collagen reduced the gene expression of inflammatory factors in MSC compared to culturing on laminin (De Lisio et al., 2014). Preconditioning of the MSC prior to administration has also been investigated, where low doses of toxic or lethal factors or exposures to hypoxic or nutrient deprived environment triggered a beneficial effect of the cells with increased expression of immunomodulatory, anti-inflammatory and repair factors (Silva et al., 2018; Nonaka et al., 2020). In a recent study, lung-derived MSCs were biophysically preconditioned to modulate their paracrine signaling by culturing the cells in a natural microenvironment, on lung scaffolds, and subjecting them to cyclic stretch prior to being injected into a rat model for acute respiratory distress syndrome (ARDS). Compared to non-preconditioned MSCs, rats treated with biophysically preconditioned MSCs had an improvement in lung elastance and reduced amounts of inflammatory cytokines in the bronchoalveolar lavage fluid (Nonaka et al., 2020). In a recent study biophysical and biochemical cues were combined to explore the immunological effect of MSC. Bone marrow-derived MSC were encapsulated in alginate-based hydrogels with different stiffnesses and pre-cultured prior to exposure to the inflammatory cytokine TNF-α. MSC preconditioned in the softer hydrogel in combination with TNFα had an improved effect in controlling monocyte turnover (Wong et al., 2020). These studies demonstrate how intrinsic cues of the microenvironment impact cellular behavior that can be harnessed in improving the therapeutic potential of MSCs.

## Future Directions

For MSC therapy to become an efficient alternative to current medicine in IPF and COPD, it is essential to ensure the functional outcome of the cells in the diseased tissue. One way is to control the microenvironment in which the cells are delivered. Recent advances have demonstrated that MSC actively interact and modify their pericellular environment, that there is an active interplay between the surrounding properties, both in terms of structural properties and chemical composition, thus will play a role in directing MSC function (Ferreira et al., 2018; Loebel et al., 2019). Several obstacles remain with current MSC therapy. Ensure long-term retention of the MSCs in the lung is one, although they do get trapped in pulmonary capillaries after systemic administration, they are considered cleared within 24 h (Wang et al., 2009). MSCs are also vulnerable to the inflammatory microenvironment, which affect their restorative potential (Silva et al., 2018). Therefore, we foresee a future, where we are able to increase the retention time in the organ and control the therapeutic effect of MSCs by providing a microenvironment that supports these properties. Research into the field of artificial scaffolds for medicinal purposes that can retain a particular therapeutic property is warranted to push forward this line of novel therapies for chronic lung diseases. Modifying the microenvironment *in situ* may hold difficulties in its approach in retaining tuned levels of cell mediators or in adapting specific ECM components in the location of transplanted MSC. Identifying key elements or biological properties of the ECM warrants further investigation as this would allow for more precise and directed manipulation of the pathological tissue *in situ*. The environmental responsiveness of the MSCs creates both challenges and opportunities in applying transplanted MSC as a cell-based therapy in lung diseases that are marked with massive structural and compositional tissue remodeling. Thus, pre-conditioning of MSC may hold a more transient approach in overcoming the powerful influential impact of surrounding tissue on cellular behavior, providing a more instant regenerative response.

The artificial scaffolds hold great promise in future therapeutic purposes as it can combine cell-instructive cues together with a supporting microenvironment, delicately designed in both structure and biomechanics, that together yield an optimal curative setting for MSC. The purpose of a controlled microenvironment is to create an interim support as well as *in situ* cell guidance over time as the scaffold is degraded and replaced with functional lung tissue. By providing structural support, we anticipate that both the MSC retention time will be greatly improved with remained activity and allowing endogenous cells to repopulate lost tissue area.

Altogether, harnessing the microenvironment holds promise to ameliorate future cell therapeutic regimens to be able to induce lung regeneration and healing of lung tissue in chronic lung diseases marked with massive structural remodeling like IPF and COPD.
